# In vitro antimicrobial susceptibility testing of human *Brucella melitensis* isolates from Ulanqab of Inner Mongolia, China

**DOI:** 10.1186/s12879-018-2947-6

**Published:** 2018-01-16

**Authors:** Zhi-guo Liu, Dong-dong Di, Miao Wang, Ri-hong Liu, Hong-yan Zhao, Dong-ri Piao, Zhong-zhi Zhao, Yong-qing Hao, Ya-nan Du, Hai Jiang, Bu-yun Cui, Xian-zhu Xia

**Affiliations:** 1 College of Veterinary Medical Inner Mongolia Agriculture University, Hohhot, 010018 China; 2 Ulanqab Centre for Endemic Disease Prevention and Control, Health and Family Planning Commission of Ulanqab, Ulanqab west Road, Jining, 012000 Inner Mongolia; 3 Laboratory of Zoonoses, China Animal Health and Epidemiology Center, MOA, Qingdao, China; 4 State Key Laboratory for Infectious Disease Prevention and Control, National Institute for Communicable Disease Control and Prevention/Collaborative Innovation Center for Diagnosis and Treatment of Infectious Disease, Chinese Center for Disease Control and Prevention, 155 Changbai Road, Changping, Beijing, 102206 People’s Republic of China; 5 Institute of Military Veterinary AMMS, Changchun, 130062 China

**Keywords:** *Brucella melitensis*, brucellosis, Antimicrobial susceptibility, Ulanqab, Inner Mongolia

## Abstract

**Background:**

Brucellosis is an endemic disease in the Inner Mongolia Autonomous Region of China and Ulanqab exhibits the highest prevalence of brucellosis in this region. Due to the complex nature of Brucellosis, a cure for this disease has proven to be elusive. Furthermore, the reduced susceptibility of *Brucella spp.* to antimicrobial agents has been reported as a potential cause of therapeutic failure. However, detailed in vitro antimicrobial susceptibility patterns pertaining to *Brucella* isolates from this region have not yet been published. The aim of this study was to evaluate the antibiotic susceptibility profile of *Brucella melitensis* clinical isolates from Ulanqab, Inner Mongolia, China.

**Methods:**

A total of 85 *B. melitesis* isolates were obtained from humans in Ulanqab of Inner Mongolia, China; the antimicrobial susceptibility of 85 clinical isolates to nine antibiotics was assessed using the E-test method according to the CLSI (Clinical and Laboratory Standards Institute) guidelines.

**Results:**

All of the tested isolates were susceptible to minocycline, sparfloxacin, doxycycline, tetracycline, ciprofloxacin, gentamicin and levofloxacin. Resistance to rifampin and cotrimoxazole was observed in 1.0% (1/85) and 7.0% (6/85) of the isolates, respectively. However, *rpoB* gene mutations were not observed in single isolates exhibiting resistance to rifampin.

**Conclusions:**

We observed that *B. melitensis* isolates are susceptible to the majority of the tested antibiotics. Furthermore, minocycline and sparfloxacin exhibited extremely high bactericidal effects in relation to the *B. melitensis* isolates. The sensitivity of commonly used drugs for the treatment of brucellosis should be regularly monitored. To the best of our knowledge, this is the first report of rifampin and cotrimoxazole resistant isolates of *B. melitensis* in China. In summary, based on the findings from this study, we suggest that antibiotic administration and use should be rationalized to prevent future drug resistance.

## Background

Brucellosis is a zoonotic disease that can cause severe morbidity in humans. This disease also results in multifarious medical challenges globally, especially in developing regions [1]. The genus *Brucella* encompasses intracellular bacterial pathogens that infect host macrophage cells [2]. Thus, associated treatment regimens are protracted and require macrophage penetration to facilitate effective treatment [3]. Therefore, only a limited number of antibiotics are effective against the causative microorganisms.

Inner Mongolia has experienced a dramatic history of brucellosis, and incidences of human brucellosis have risen rapidly since 2010 [4]. Ulanqab exhibits the highest prevalence of brucellosis in Inner Mongolia. Between 2011 and 2015, the average annual incidence of brucellosis was 57/100,000. In this study, almost all of the 85 patients analyzed had a history of exposure to sheep, and contact with infected domestic animals (sheep) was the most likely source of infection. Furthermore, we speculate that contaminated animal produce (e.g., undercooked meat and unpasteurized milk) is also as an important source of infection. However, misdiagnosis and mistreatment are widespread due to the fact that brucellosis patients typically lack obvious signs and symptoms of infection [5, 6]. In the majority of cases, therapeutic failure is caused by inadequate therapeutic regimens. Although resistant strains are rarely caused by therapy failure, resistance to commonly used antimicrobial agents may lead to treatment inhibition. Consequently, a human brucellosis diagnostic and treatment project was initiated by the Department of Health of the Inner Mongolia autonomous region in 2011. This program aimed to normalize brucellosis diagnosis and treatment, and improve the regimens used to treat brucellosis, thereby ameliorating problems relating to brucellosis chronicity. The project detailed which antibiotics should be chosen to treat the bacterial infective agents that cause brucellosis. This project also published recommendations regarding which antibiotic combinations should be utilized for infections. Unfortunately, detailed in vitro antimicrobial susceptibility patterns pertaining to *Brucella* isolates from this region have not yet been published. Furthermore, some studies have also shown that drug regimen recommendations published by WHO are not always implemented in clinical practice and relapses due to inadequate therapy occurs in 5–10% of patients [7]. Consequently, sporadic cases of human *Brucella* isolates with antibiotic resistance [8, 9] and human *Brucella* isolates that are resistant to rifampin and cotrimoxazole have been reported [10, 11]. Thus, the main objective of this study was to determine the antimicrobial susceptibility profile of human *Brucella* isolates. It is hoped that this information will provide a platform that will help to elucidate the most optimal regimens for brucellosis treatment, thereby facilitating the discovery of more effective therapeutic drug treatment strategies for brucellosis treatment.

## Methods

### Bacterial isolates

A total of 85 *Brucella* isolates obtained from patient blood samples (*n* = 85) were collected between March 2011 and September 2015 in Ulanqab, Inner Mongolia. The identification of isolates was based on standard procedures [12], and included analysis of colony morphology, Gram stain reaction, CO_2_ requirements, H_2_S production, inhibition of growth by basic Fuchsin and Thionin, agglutination with monospecific antisera and phage lysis testing. Species-level identification was undertaken using *B. abortus*, *B. melitensis*, *B. ovis*, *B. suis* PCR (AMOS-PCR) [13]. All cultures were processed in a Biological Safety Laboratory III.

### Antimicrobial susceptibility testing

In vitro evaluations of antibiotic efficacy against *B. melitensis* were based on the Minimal Inhibitory Concentration (MIC) values. The MIC’s (MIC_50_ and MIC_90_) of rifampin, doxycycline, gentamicin, tetracycline, ciprofloxacin, levofloxacin, minocycline, sparfloxacin and cotrimoxazole to *Brucella melitensis* were determined using the E-test strip method (E-test strips, Wenzhou kont biology and technology, Ltd., China) according to the CLSI guidelines. The plates were incubated in ambient air at 35 °C and evaluated after 48 h. MIC breakpoints of levofloxacin, tetracycline, doxycycline and cotrimoxazole were used as recommended by CLSI [14]. Because MIC breakpoints for *Brucella* against rifampin, ciprofloxacin and other antibiotics have not yet been established, guidelines for slow-growing bacteria (*H. influenzae*) were used as an alternative [15]. The MIC was interpreted as the value at which the inhibition zone intercepted the scale on the E-test strip. MIC_50_ and MIC_90_ levels were defined as the lowest concentration of the antibiotic at which 50% and 90% of the isolates were inhibited, respectively. The reference strains, *B. melitensis* 16 M and *H. influenza* ATCC 10211I*,* were used as quality control strains.

### PCR assay of the *rpoB* gene

Two hot spots in the *rpoB* (RNA polymerase, beta subunit) gene along with the whole *rpoB* gene were amplified and sequenced for isolates (*n* = 3) that showed an elevated MIC value to rifampin (≥ 2 μg/mL), as described previously [16, 17]. Sequence and data analysis were conducted as previously described [16].

## Results

The MIC range, MIC_50_ and MIC_90_ values of *Brucella melitensis* are shown in Table 1. All of the tested isolates were susceptible to minocycline (MIC_90_, 0.032 μg/mL) and sparfloxacin (MIC_90_, 0.032 μg/mL). In addition, doxycycline (MIC_90_, 0.25 μg/mL), gentamicin (MIC_90_, 1 μg/mL), tetracycline (MIC_90_, 0.5 μg/mL), ciprofloxacin (MIC_90_, 0.5 μg/mL) and levofloxacin (MIC_90_, 0.5 μg/mL) showed 100% susceptibility when MIC breakpoint criteria for *H. influenzae* [14] were used. Rifampin MIC values ranged from 0.5 to 2 μg/mL and when compared with MIC breakpoint criteria for *H. influenzae* [14], resistance (MIC ≥2 mg/L) was demonstrated in 1.0% (1/85) of the analyzed strains. Fifty-nine strains were susceptible to rifampin, twenty-five strains exhibited intermediate-resistance to rifampin and one strain was resistant to rifampin. After sequencing of the *rpoB* gene, no mutations were observed. The cotrimoxazole MIC values ranged from 0.032 to 2 μg/mL (MIC_90_, 1.0 μg/mL). Six strains exhibited resistance to cotrimoxazole. No association between biovar type and the susceptibility profile of the tested *B. melitensis* strains was observed. The MIC values (μg/mL) of 85 *B. melitensis* strains are shown in Table 2.

**Table 1:** MIC range and MIC_50_ and MIC_90_ values of nine antimicrobial agents

Antibiotic	Range (μg/mL)	MIC_50_ (μg/mL)	MIC_90_ (μg/mL)	(S)	(I)	(R)	Breakpoint for Susceptibility (μg/mL)
Minocycline	0.032	0.032	0.032	85	–	–	≤0.5^a^
Sparfloxacin	0.032	0.032	0.032	85	–	–	≤0.5^a^
Doxycycline	0.032–0.25	0.125	0.25	85	–	–	≤1
Tetracycline	0.064–1.0	0.25	0.5	85	–	–	≤1
Ciprofloxacin	0.125–1.0	0.5	0.5	85	–	–	≤1^a^
Levofloxacin	0.125–1.0	0.5	0.5	85	–	–	≤1
Gentamicin	0.25–1.0	0.5	1.0	85	–	–	≤4^a^
Rifampin	0.5–2.0	0.5	1.0	59	25	1	≤2^a^
Cotrimoxazole	0.032–2.0	0.5	1.0	79	–	6	≤2/38

^a^CLSI breakpoints for slow-growing bacteria (*Haemophilus spp.*)

**Table 2:** The MIC values (μg/mL) for *Brucella melitensis* strains (*n*: 85)

Antibiotics	MIC results (μg/mL)							
	0.032	0.064	0.125	0.25	0.5	1	2	R
Minocycline	85							
Sparfloxacin	85							
Doxycycline	3	17	24	41				
Tetracycline		15	21	16	30	3		
Ciprofloxacin			2	34	47	2		
Levofloxacin			5	24	52	4		
Gentamicin				15	55	15		
Rifampin					59	23	2	1
Cotrimoxazole	7	16	11	10	12	21	2	6

## Discussion

In the present study, we investigated the in vitro susceptibility of 85 *B. melitensis* strains isolated from humans in Ulanqab (where human brucellosis is endemic) to a variety of antibiotics that are commonly used in brucellosis treatment including rifampin, doxycycline, levofloxacin, tetracycline. In addition, two new antibiotics, sparfloxacin and minocycline, were also evaluated (Table 1). All of the isolates were susceptible to all of the analyzed antibiotics apart from rifampin and cotrimoxazole. A total of 1% (1/85) of the analyzed strains were resistant to rifampin and 7.0% (6/85) of the isolates were resistant to cotrimoxazole. These results are similar to those previously reported. Abdel Maksoud et al. reported that 64% of *Brucella* isolates from Egypt were resistant to rifampin [18]. Similarly, Lopez-Merino et al. reported that rifampin was less effective than other antibiotics against *Brucella* strains, and cotrimoxazole showed the poorest activity with an MIC_90_ of 8.0 mg/mL [19]. Furthermore, Ilhan et al. also reported a resistance rate of 9.7% to rifampin for *B. melitensis* and the highest resistance rate (46.3%) was observed for cotrimoxazole [20]. China is the world’s most populous country and tens of thousands of tons of antibiotics are used annually. Recent research has revealed that the total usage of antibiotics in China has reached 162,000 tons; this is almost equal to half of the total global usage of antibiotics. In 2013, approximately 7890, 6950, and 22,500 tons of sulfonamides, tetracyclines, and quinolones were used in China, respectively. The amount of rifampin used in 2013 remains unknown; however, it is not likely to be less than the any of the afore-mentioned antibiotics.

Therefore, in order to better understand the use of antibiotics in China this current study assessed a total of nine antibiotics; these antibiotics are the most commonly used drugs in the treatment of brucellosis and should be regularly monitored in relation to drug susceptibility.

Rifampin belongs to the rifamycin group of antibiotics and elicits its bactericidal effects by blocking the synthesis of bacterial RNA and protein [21]. In the present study, 96.5% (82/85) of *Brucella* strains with MIC ≤ 1 μg/mL for rifampin were considered susceptible according to the CLSI breakpoints for slow-growing bacteria. Two *Brucella* strains exhibited higher MIC’s of rifampin (≥2 μg/mL) and one strain was resistant to rifampin. Interestingly, higher MIC’s of rifampin have been reported in relation to a significant proportion of strains in Egypt (64%) [18], Malaysia (70%) [22] and Brazil (36.73%) [10]. Furthermore, some of the strains that were shown to be resistant to rifampin were also observed in previous studies that were performed in Turkey (9.7%) [20] and Brazil (2.04%). However, the impact of high MIC’s on clinical outcomes is not yet clear and further research is required to elucidate the impact of the latter. Furthermore, a large number of patients cannot use rifampin over extended durations due to adverse gastrointestinal reactions. In order to ensure effective treatment, our hospital started using rifamycin sodium as a replacement for rifampin from 2000. At present, rifamycin sodium drug sensitivity test strips are not available on the market. Thus, this study did not investigate bacterial susceptibility with respect to rifamycin sodium. In a separate retrospective cohort study, a combination of doxycycline and streptomycin was found to be the preferred regimen followed by co-administration of doxycycline and rifampin; no relapse or therapeutic failures were detected following these regimens [23]. However, because rifampin can enhance the plasma clearance of doxycycline resulting in lower doxycycline levels [24], we suggest that alternative medicines or combinations of antibiotics should be adopted to improve curative effects and reduce patient discomfort.

Mutations conferring rifampin resistance are confined almost exclusively to the *rpoB* gene in most organisms. These mutations result in a decreased affinity of the DNA-dependent RNA polymerase to rifampin. However, alternative mechanisms of rifampin resistance have also been reported [25]. We did not observe any mutations in the *rpoB* gene of isolates that exhibited high MIC’s or isolates that were resistant to rifampin. A previous study reported more than 100 rifampin resistant *B. melitensis* isolates in Qatar. However, *rpoB* gene sequencing, results revealed that no *rpoB* mutations were found among those isolates [26]. Moreover, because both brucellosis and tuberculosis are endemic in the analyzed region and rifampin is a first-line anti-tuberculosis agent, widespread use of rifampin has most likely resulted in higher MIC’s pertaining to this antibiotic agent. Accordingly, we suggest that rifamycin sodium should be considered as a viable alternative for brucellosis treatment in regions exhibiting higher rates of rifampin-resistance. This study confirmed that *rpoB* gene mutations were not present in the isolates that were resistant to rifampin. However, the previous study confirmed that *rpoB* gene mutations were not the only mechanisms underpinning *Brucella* resistance to rifampin. Indeed, the excitation of several metabolic processes can also contribute to rifampin resistance in *Brucella* [27]. Furthermore, *Brucella* spp*.* share similar genomes and harbor two circular chromosomes that contain a number of putative drug efflux transporters [28, 29]. Consequently, efflux transporters may be involved in rifampin-resistance mechanisms in *Brucella*.

Cotrimoxazole is an alternative antimicrobial agent that has been recommended for the treatment of brucellosis in pregnant females and children. However, the development of resistance to cotrimoxazole in *B. melitensis* has become an important issue. In the present study, six isolates were resistant to cotrimoxazole. This finding is in agreement with previous studies [11, 19]. Moreover, Irajian et al. also reported that two *Brucella* strains were completely resistant to cotrimoxazole and one isolates exhibited intermediate resistance to it [30]. Sulfonamide resistance in most Gram-negative species involves the acquisition of an additional deoxyhypusine synthase (DHPS) gene on either a plasmid or a transposable element. The latter gene encodes for an enzyme that is not inhibited by sulfonamide and therefore bypasses inhibition of the chromosomally-encoded enzyme [31, 32]. Whole genome sequencing of drug-resistant strains is required to elucidate the mechanisms that underlie cotrimoxazole resistance. Furthermore, continuous surveillance programs should be implemented in hospital and clinical settings to better control and treat brucellosis in pregnant females and children.

A previous study reported that sparfloxacin, a new generation fluoroquinolone, exhibits excellent activity against *Brucella*. In our study, this drug exhibited excellent antimicrobial activity against all of the species analyzed. Minocycline, a second generation broad-spectrum antibiotic, displayed the strongest antibacterial activity and has a relatively long half-life, with plasma concentrations that were two to four times greater than those for other drugs [33]. Furthermore, this antibiotic has the greatest central nervous system (CNS) penetrance when compared with other tetracyclines, and the lipophilicity of minocycline appears to facilitate a high degree of intestinal absorption and tissue penetration [34]. We suggest that sparfloxacin and minocycline can be used as first-line antibiotics for the treatment of relapses and complications pertaining to patients with brucellosis. However, further studies are required to determine the role of these two antibiotics in the treatment of brucellosis.

Among the 85 *B. melitensis* patients that were analyzed as part of this study, 50 patients received oral drug therapy (rifapentine and tetracycline: 6 weeks) and 35 cases required hospitalization (intravenous injection of rifamycin sodium, levofloxacin and cefoperazone sulbactam: 2 weeks) following diagnosis with *Brucella* infections. Following administration of medication for a further two weeks, nearly all of the patients improved to different degrees. Approximately six months later, one of the previously hospitalized patients was re-admitted to the hospital. Following an interview with this patient, it transpired that after treatment the patient continued to engage in sheep farming. Thus, it was suspected that the latter individual suffered a relapse. To determine the prevalence of relapse occurrence following the associated treatment regimen, a greater number of cases need to be analyzed. Another isolate from a different patient (a student) showed resistance to rifampin. The clinical manifestations (lumbago and joint pain) of this patient were relieved following the administration of rifapentine and tetracycline for 6 weeks. However, eight months after drug withdrawal, lumbago symptoms began to re-occur. This demonstrated that the patient was not completely cured and a timely review of the treatment regimen was required.

## Conclusions

In summary, most of the antibacterial agents tested in our study demonstrated activity against *Brucella melitensis* and could be used in therapeutic regimens to combat associated infections. The exceptions were rifampin and cotrimoxazole. However, the intracellular localization of *Brucella* within monocytes and macrophages (of the reticulo-endothelial system) limits the choice of antimicrobial agents that can be used for the effective treatment of systemic and localized brucellosis. Although a variety of antimicrobial agents appear to be active in vitro, the results of susceptibility testing do not always correlate with clinical efficacy [35]. Thus, the specific conditions pertaining to each patient and the experience of the clinicians often determine which antibiotics are selected for treatment. However, in vitro sensitivity data raise the possibility of monitoring resistance in the future. Regular monitoring of drug sensitivity in *Brucella* is essential, and antibiotic administration and use should be rationalized to prevent future drug resistance.

## Abbreviations

CLSI: Clinical and Laboratory Standards Institute
CNS: central nervous system
DHPS: deoxyhypusine synthase
MIC: Minimal Inhibitory Concentration
*rpoB*: RNA polymerase, beta subunit
